# Neurotransmitters in hiccups

**DOI:** 10.1186/s40064-016-3034-3

**Published:** 2016-08-17

**Authors:** Fauzia Nausheen, Hina Mohsin, Shaheen E. Lakhan

**Affiliations:** 1 Department of Medical Education, California University of Science and Medicine - School of Medicine, 1405 W. Valley Blvd, Suite 101, Colton, CA 92343 USA; 2 Department of Neurology, California University of Science and Medicine - School of Medicine, Colton, CA USA

**Keywords:** Hiccup, Neurotransmitters, Therapies

## Abstract

Hiccups are the sudden involuntary contractions of the diaphragm and intercostal muscles. They are generally benign and self-limited, however, in some cases they are chronic and debilitating. There are approximately 4000 admissions for hiccups each year in the United States. The hiccup reflex arc is composed of three components: (1) an afferent limb including the phrenic, vagus, and sympathetic nerves, (2) the central processing unit in the midbrain, and (3) the efferent limb carrying motor fibers to the diaphragm and intercostal muscles. Hiccups may be idiopathic, organic, psychogenic, or medication-induced. Data obtained largely from case studies of hiccups either induced by or treated with medications have led to hypotheses on the neurotransmitters involved. The central neurotransmitters implicated in hiccups include GABA, dopamine, and serotonin, while the peripheral neurotransmitters are epinephrine, norepinephrine, acetylcholine, and histamine. Further studies are needed to characterize the nature of neurotransmitters at each anatomical level of the reflex arc to better target hiccups pharmacologically.

## Background

The term “singultus” (hiccup) comes from singult; a Latin word that means ‘sob’ or ‘gasp’. It refers to the sounds that are produced by the sudden involuntary contractions of the diaphragm and intercostal muscles followed by an abrupt contraction of the glottis. The air strikes the closed glottis and results in the characteristic “hiccup” sound. Hiccups are usually benign and self-limiting. They generally start without any specific reason and disappear in a few minutes. Brief episodes of hiccups are common in healthy individuals after a large meal, intake of alcoholic beverages, or sudden excitement.

This article provides a review of different neurotransmitters that are related in the mechanism of action of the most commonly used drugs to treat hiccups, and the medications that induced hiccups. At the end, this paper draws a conclusion about the neurotransmitters involved in the pathophysiology of hiccups.

## Epidemiology

The classification of hiccups is based on their duration. An acute attack lasts less than forty-eight hours. Persistent hiccups last more than 2 days. Intractable hiccups are present if the attack lasts more than 1 month. Persistent hiccups are most likely to be associated with an underlying pathological, anatomic or organic disease process (Cymet 2002). Intractable hiccups that continue for more than 1 month are usually indicative of a serious organic disturbance (Vaidya 2000). If left untreated, intractable hiccups can cause severe discomfort, depression, reduced physical strength, and even death (Consults 2011). According to a report by William H. Dobelle, approximately 4000 hospital admissions due to hiccups are reported each year in the United States (Dobelle 1999). The intractable hiccups are more common in men (82 %) than in women. Most of the men suffering from hiccups are 50 years of age or older (Cymet 2002). Psychogenic hiccups have been reported to occur more commonly in women. The usual rate for hiccups is four to sixty per minute with fairly constant frequency in an individual (Howes 2012). Pathological hiccups can be explained as a form of epilepsy or a failure of supra-spinal inhibition (Launois et al. 1993; Lewis 1985). The incidence and prevalence of persistent and intractable hiccups in the community has not been studied.

## Pathophysiology

**Fig. 1:**
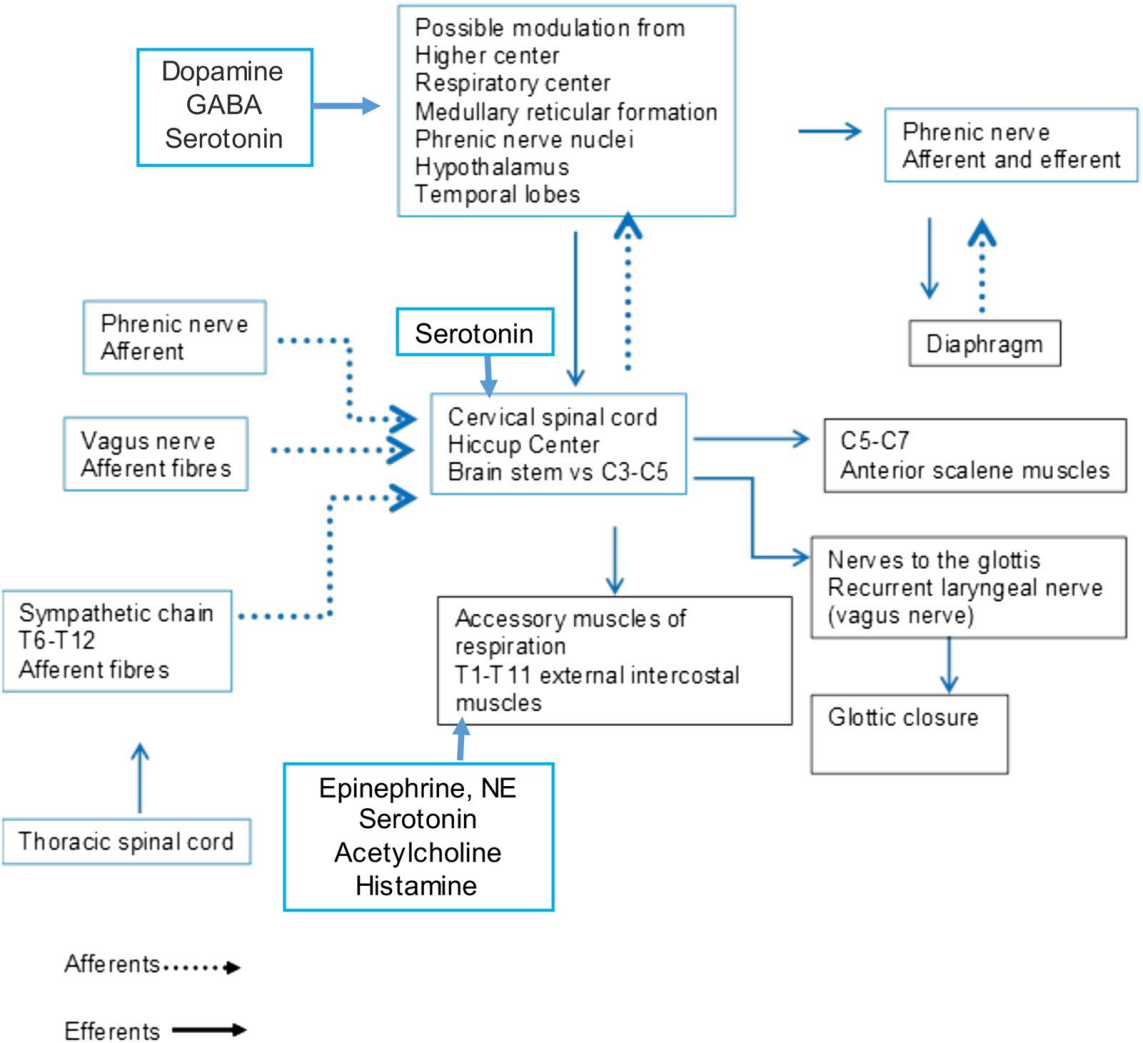
Hiccup reflex arc with neurotransmitters

The pathophysiological mechanism of hiccups is related to lesions in its reflex arc as shown in Fig. 1. The reflex arc is comprised of three components:

1. The afferent limb including phrenic, vagus, and sympathetic nerves to pass on somatic and visceral sensory signals;
2. The central processing unit in the midbrain; and
3. The efferent limb travelling in motor fibers of phrenic nerve to diaphragm and accessory nerves to the intercostal muscles, respectively.

The central component is located in the periaqueductal grey, subthalamic nuclei (Hansen and Rosenberg 1993) among the brain stem respiratory center, phrenic nerve nuclei, reticular formation and hypothalamus. The central component for hiccups lies in the medulla and is thought to be entirely separate from the pathways involved in rhythmic breathing (Davis 1970). Dopamine, gamma-amino-butyric-acid (GABA), serotonin, glutamate, and glycine neurotransmitters can regulate this central mechanism. The hiccup arc has modulatory input from catecholaminergic and serotonergic afferents.

The release of 5-hydroxyl-tryptamine (5HT) from the gut enterochromaffin cells and enteric vagal afferents may also lead to hiccups as seen in a case report following administration of cisplatinum, a chemotherapeutic agent (Jatoi 2009). The mental branch of the trigeminal nerve was also postulated to develop hiccups when stimulated via chin shaving (Todisco et al. 2004). Significant negative intrathoracic pressure may occur during hiccups that may result in hypotension, bradycardia, pneumomediastinum, and subcutaneous emphysema (Rousseau 1995). The mechanism of action of hiccups might be mediated through agonizing 5-HT1A and antagonizing 5-HT2A receptors to enhance the activity of the phrenic nerve, thereby inducing hiccups. This concept was supported in a case report in which quetiapine was successfully used to resolve the aripirazole-induced hiccups. This may suggest the partial agonist and relatively high 5-HT1A receptors binding affinities in the pathophysiology of hiccups (Gilson and Busalacchi 1998). It is postulated in a study that the GABA-containing cells in the nucleus raphe are the source of GABA-nergic inhibition of the hiccup center (Musumeci et al. 2000).

The most commonly used pharmacological treatments include metoclopramide which reduces the intensity of esophageal contraction, chlorpromazine (Twycross et al. 2002), baclofen, nifedipine which reverses the abnormal depolarization in the hiccups reflex, valproic acid that enhances GABA transmission centrally (Smith and Busracamwongs 2003), antipsychotics, glucagon, GABA analogue which acts by activating an inhibitory neurotransmitter, and dimethlamine derivative of phenothiazine which acts centrally by dopamine blockade in the hypothalamus (Friedman 1996). Baclofen (GABA-agonist) is among the substances that act through the nervous system and has by far the best ability to treat chronic hiccups (Guelaud et al. 1995; Oshima et al. 1998; Petroianu et al. 1997; Steger et al. 2015). However, a Cochrane systemic review found insufficient evidence as to which pharmacological agent is best for hiccups (Moretto et al. 2013). Hiccups are the manifestation of diaphragmatic myoclonus and are considered to be a form of physiologic myoclonus.

## Etiology

In the review of recent literature, a variety of hiccups etiologies have been reported (idiopathic, organic, psychogenic, and medication-induced) induced have been reported. Table 1 provides an overview of pathology that has been reliably linked to this condition. It has been suggested that damage to the cervical cord, brainstem, hypothalamus, and supra-tentorial area precipitate hiccups by stimulating the hiccups reflex arc or decreasing the normal inhibition of hiccup neurons. It is suggested that all potentially successful therapeutic drugs used to treat hiccups either decrease the input from gastrointestinal tract (GIT) to the hiccups center or decrease the excitability and output from the hiccups center (Burke et al. 1988; Petroianu et al. 1997).

**Table 1:** Common causes of hiccups

Central nervous system	Vascular	Stroke, Infarct, SLE related vascular disorders and aneurysm
	Infectious	Meningitis and Encephalitis
	Structural	Brain injury, Intracranial tumors
	Inflammation	Neuromyelitis optica and multiple sclerosis
	Miscellaneous	Seizure, Parkinson’s Syndrome
Peripheral Nervous System (phrenic, vagal and sympathetic nerves)	Gastrointestinal	Hiatus hernia, Esophageal cancer, Gastro-esophageal reflex disease, stomach volvulus and H.pylori infection, Pancreatitis, Abdominal abscess and Abdominal tumors
Thoracic	Cardiovascular	Myocardial Ischemia, Pericarditis, Thoracic Aneurysm
	Pulmonary	Bronchitis, Pneumonia, Asthma, Bronchial carcinoma, Tuberculosis
Ear, Nose and Throat		Rhinitis, Otitis, Pharyngitis, Foreign body in nose or ear
Other causes	Toxic metabolic	Electrolyte imbalance, Alcohol, Chronic renal failure, Diabetes mellitus
	Pharmacological	Steriods, benzodiazepines, chlordiazepoxide, diazepam, antibiotics, sulfonamides, opioids, cisplatinum (Jatoi 2009), analeptic agent, Methyldopa, l-dopa, Dopamine
	Psychosomatic	Anxiety, sleep deprivation, stress and fear

## Neurotransmitters targets in hiccups

The exact etiology of hiccups is unclear, and it is unknown why diverse drugs like dopamine blocking agents (DBA), baclofen, clonazepam, and phenytoin, which have widely varying mechanisms of action, can be effective in the treatment of hiccups (Peleg and Peleg 2000). Table 2 summarize drugs which induce and treat hiccups and their potential neurotransmitters.

**Table 2:** List of drugs which induce and treat hiccups and their potential neurotransmitters

Location	Neurotransmitters	Drug-induced hiccups	Treatment of hiccups
Central neurotransmitters; Respiratory center, medullary reticular formation, phreni**c nerve nuclei, hypothalamus, temporal lobes	Gama-amino butyric acid (GABA)	Propofol (Jones et al. 1995)	Valproic acid (Smith and Busracamwongs 2003)
		Benzodiazepine (Jones et al. 1995)	Baclofen (Guelaud et al. 1995; Oshima et al. 1998; Petroianu et al. 1997; Smith and Busracamwongs 2003; Zhang et al. 2014)
		Barbiturates (MacDonald et al. 1989)	Gabapentin (Petroianu et al. 1998, 2000; Moretti et al. 2004; Liang et al. 2005; Porzio et al. 2010)
			Midazolam (Smith and Busracamwongs 2003)
	Dopamine	Aripiprazole (Ray et al. 2009)	Metaclopramide (Smith and Busracamwongs 2003; Stav et al. 1992; Bateman 1983)
			Chlorpromazine (Smith and Busracamwongs 2003)
		Dopamine agonists (pirebedil, pergolide, pramipexo) (Sharma et al. 2006; Lester et al. 2007)	Baclofen (Martinez-Ruiz et al. 2004)
			Clonazepam (Martinez-Ruiz et al. 2004)
		Levodopa (Luquin et al. 1992)	Phenytoin (Martinez-Ruiz et al. 2004)
			Pramipexol (Vaidya 2000; Martinez-Ruiz et al. 2004)
	Serotonin		Olanzapine (Alderfer and Arciniegas 2006)
			Amantadine (Wilcox et al. 2009; Askenasy et al. 1988)
			Sertraline (Vaidya 2000)
			Tandospirone (Takahashi et al. 2004)
			Risperidone (Nishikawa et al. 2015)
Peripheral neuro transmitters; diaphragm, glottis, scalene muscle, respiratory muscle, GIT	Histamine		Omeprazole (Petroianu et al. 1997)
	Epinephrine Norepine-phrine		Methylphenidate (Marechal et al. 2003; Pollock et al. 2009)
	Acetylcholine		Metoclopramide (Butterworth IV et al. 2005; Smith and Busracamwongs 2003)

### Gama-amino butyric acid (GABA)

There is strong evidence that the GABA is one of the neurotransmitters involved in the hiccups reflex at the central level. GABA functions as an inhibitory mediator at the interneuron level in the brain and in the spinal cord (presynaptic inhibition) by altering trans-membrane potential. Glutamic acid is decarboxylated to produce GABA by the enzyme l-glutamate decarboxylase (Rodwell 2012). The inhibition of synapses by GABA has been shown in the cerebellar cortex, hippocampus, olfactory bulb, cuneate nucleus, caudate nucleus, substantia nigra, septal nucleus, and between the vestibular and trochlear motor neurons. There are three main types of GABA receptors—GABA-A, -B, and -C. GABA-A are the most abundant receptors and the site of action of many neuro active drugs such as benzodiazepines, barbiturates, ethanol and volatile anesthetics (Molinoff 2011). Valproic acid enhances GABA transmission centrally (Smith and Busracamwongs 2003) and is one of the most commonly suggested therapies for hiccups. It is postulated in a study that the GABA-containing cells in the nucleus raphe are the source of GABA-ergic inhibition of the hiccup center (Musumeci et al. 2000).

### Dopamine

There are many studies to support the convincing role of dopamine as the central neurotransmitter involved in hiccup pathogenesis. Dopamine is found in the brain and peripherally in the adrenal medulla, plexuses of the GI tract, and the enteric nervous system. Dopamine is an adrenergic agonist that is non-selective (Morgan et al. 2005). There are five dopamine receptor subtypes that have been delineated by pharmacological analysis that are the bases of a subtype of selective drugs. There are three major dopaminergic pathways in the central nervous system:

1. the niagrostriatal pathway which is important in Parkinson’s disease;
2. the mesolimbic pathway which plays a role in psychiatric disorders; and
3. the tuberoinfundibular pathway, which is related to the regulation of the endocrine system.

Dopamine is an immediate precursor of norepinephrine. The synthesis of dopamine can be increased by giving DOPA (Smith and Busracamwongs 2003; Ashley and Krych 1995). There are many studies, in which patients have been treated successfully by using dopamine blocking drugs such as metochlopromide and chlorpromazine (Smith and Busracamwongs 2003) which supports the evidence that dopamine acts as the central neurotransmitter.

### 5-Hydroxyl tryptamine (5HT; serotonin)

Many studies support the role of 5HT (serotonin) as the neurotransmitter of hiccups. Serotonin is an important central nervous system neurotransmitter and a local hormone. It is also found in high concentrations in enterochromaffin cells throughout the GIT that are the site of synthesis and storage of 5 HT from tryptophan. It regulates the smooth muscles in the GIT to increase the tone and facilitate the peristalsis via 5HT2 receptors (Katzung 2012). It is a powerful vasoconstrictor mainly through 5HT2 receptor but it dilates the blood vessels of the heart and skeletal muscles (Katzung 2012). It is produced in two steps: in step 1, tryptophan in converted to 5HT and in the next step, the 5HT is converted to serotonin. Increasing the concentration of tryptophan in the brain can increase the high concentration of 5HT and serotonin. Drugs that increase serotonin levels were most commonly used as appetite suppressants. They regulate the smooth muscles in the GIT and cardiovascular system. There are fourteen subtypes of receptor of 5HT recognized. There are the 5HT1 subfamily and 5HT2 subfamilies of receptors. 5HT3 subfamilies were originally described in the periphery. There are fourteen subtypes of receptor of 5HT recognized. Cases of intractable hiccups have been successfully treated with olanzapine (Alderfer and Arciniegas 2006). Olanzapine has a complex pharmacology and its major effect is to antagonize the postsynaptic serotonergic receptors. Similarly, another case was reported describing the successful treatment of hiccups with sertraline, a selective serotonin reuptake inhibitor (SSRI), for the first time (Benowitz 2012; Vaidya 2000). In one case study, intractable hiccups were treated successfully with a tandospirone, a new anxiolytic and 5HTA1 receptor agonist (Takahashi et al. 2004). Interestingly, a case report of intractable hiccups that was refractory to the typical antipsychotic haloperidol, however, responsive to atypical antipsychotic risperidone which acts on 5HT2A, 5HT1A, 5HT1C, 5HT1D, and D2 receptors (Nishikawa et al. 2015). All these studies are evidence that serotonin is involved in the pathophysiology of hiccups.

### Histamine

Histamine is produced by decarboxylation of histadine, an amino acid, by the enzyme histadine decarboxylases (Biomedical Aspects of Histamine 2011).

Histamine is released by the various triggers from mast cells and basophils and has various biological effects through four receptors H1R, H2R, H3R, H4R. Histamine causes smooth cell contractions, vasodilatation, and gastric acid secretion. Hyperhistaminemia can cause an anaphylactic reaction (Hershko et al. 2001). There is evidence for the use of H2-receptor blockers and proton pump inhibitors (i.e. omeprazole) for the treatment of hiccups thought to act via decreasing the input from the GIT to the hiccup center (Petroianu et al. 1997).

### Epinephrine and norepinephrine (NE)

Noreepinephrine (NE) is the neurotransmitter of the post ganglionic autonomic nervous system that is involved in the reduction of gastrointestinal motility. NE is synthesized from tyrosine that is produced from phenyle alanine. Dopamine is converted to NE by dopamine beta hyroxylases. Norepinephrine is converted to epinephrine by the enzyme phenyle ethanolamine-N-methyltransferase. The action of neurotransmitter catecholamines is terminated by the re-uptake and metabolism by the enzymes monoamine oxidases (MOA) and catecolaminotransferases (COMT). The inhibitors of these enzymes are used in the treatment of Parkinson’s disease. Adreno receptors have four subtypes, alpha1, alpha2, and beta1 for NE mainly and B2 is for epinephrine mainly. There are studies about the successful treatment of hiccups with methylphenidate (a psycho-stimulant) generally used in attention deficit disorders (Marechal et al. 2003; Pollock et al. 2009). The methylphenidate is considered as the receptor modulator of dopamine and norepinephrine. Therefore relationship may exist between norepinephrine and epinephrine to the pathophysiology of hiccups.

### Acetylcholine

Acetylcholine is a naturally occurring neurotransmitter for the muscarinic receptors. It is a neurotransmitter of all the preganglionic autonomic fibres, most postganglionic parasympathetic, and a few post-ganglionic sympathetic fibers (Westfall and Westfall 2011). Metoclopramide, an anticholinergic, is recommended for the symptomatic relief of hiccups. There are studies that show that metoclopramide acts peripherally to reduce the intensity of esophageal contraction (Consults 2011). It has also been observed that metoclopramide have been used to treat hiccups (Smith and Busracamwongs 2003) which may explain the relation of acetylcholine as the peripheral neurotransmitter involved in the pathophysiology of hiccups.

## Conclusion

Largely through case studies involving medications, we implicate the neurotransmitters involved in hiccups. The reflex arc is potentially mediated by central neurotransmitters (GABA, dopamine, and serotonin) and peripheral neurotransmitters (epinephrine, norepinephrine, acetylcholine, and histamine). Lesions across any of these neuroanatomical structures, for instance, from the brainstem down to the diaphragm, may be responsible for the development of hiccups. Further research is needed to characterize the neurotransmitters involved in hiccups for potential new therapeutic targets.
